# Research on a new process of reconstituted landess goose steak

**DOI:** 10.1016/j.fochx.2024.102118

**Published:** 2024-12-30

**Authors:** Saisai Zhang, Hanrui Wang, SiYuan Li, Junjie Zhang, Linwu Zhuang, Shengfu Li

**Affiliations:** a School of Ocean Food and Biological Engineering, Jiangsu Ocean University, Lianyungang, China; b School of Physics and Astronnmy, The University of Edinburgh, UK; c Jiangsu Institute of Marine Resources Development, Jiangsu Ocean University, Lianyungang, China

**Keywords:** Reconstituted goose steak, Formula, Sensory score, Process research

## Abstract

The primary product currently sold from *Anser cygnoides* is foie gras, with limited research conducted on the processing of *Anser cygnoides* meat, which consequently restricts its added value. Therefore, the objective of this research is to develop a processing technique for reconstituted goose cutlets using *Anser cygnoides* meat as the main ingredient, aiming for a compact structure and intact shape after frying. The study examined the effects of compound enzyme quantity, tumbling duration, and molding time on the quality of the reconstituted goose cutlets, utilizing bonding strength, cooking loss rate, color, chewability, adhesiveness, and sensory evaluation as key metrics. Through single-factor and response surface tests, the optimal process parameters were determined as follows: a compound enzyme addition of 3.5 %, a tumbling time of 4 h, and a forming time of 10 h. Under these conditions, the bonding strength measured 34.950 g/cm^2^, and the reconstituted goose cutlet exhibited strong cohesion while maintaining its shape after frying.

## Highlights

• Processing technology of restructured goose steak with goose meat as raw material.
• Maximizing economic value by utilizing post foie gras raw materials.
• Recombinant technology facilitates the integration of meat with various materials.
• Preparation of a High Value-Added Product from Minced Meat of the Lander goose.

## 1 Introduction

China is rich in genetic resources for geese, featuring 31 indigenous breeds alongside several imported European varieties (Chen et al., 2024). Currently, the predominant sales of Lande geese in China focus on fat foie gras, with limited research conducted on the processing of *Anser cygnoides* meat. As a result, a significant quantity of by-products, including meat pieces, minced meat, and meat granules, remains underutilized (Thakur et al., 2023), which has impeded the enhancement of the added value of Lande geese (Peng & Wang, 2008). Currently, the three largest breeding bases in China are located in Linqu County, Weifang City, Shandong Province; Huoqiu County, Lu'an City, Anhui Province; and Yutai County, Jining City, Shandong Province. Linqu and Huoqiu are renowned for fattening, liver production, and deep processing, while Yutai is recognized for breeding, hatching, and brooding. The population of Lande geese in Yutai County is maintained at approximately 350,000 to 500,000 per year, with an annual brood quantity ranging from 8 to 12 million birds. This makes Yutai the leading region in the country for both the stock of Lande geese and annual brood quantity. The sales value of goose broodstock alone can reach between 160 million and 240 million Yuan (Gao et al., 2024). As the world's largest producer and consumer of broiler geese, China's consumption of goose products has been steadily increasing each year (FAO-STAT, 2020). Poultry meat is recognized for its nutritional advantages over red meat, serving as a desirable source of monounsaturated fats and polyunsaturated fatty acids while containing no trans fats, which are often found in beef and lamb (Ismail & Joo, 2017). Goose ranks as the third largest type of poultry, with its meat being particularly rich in unsaturated fatty acids (FAO-STAT, 2020; Liu & Zhou, 2013). The meat of *Anser cygnoides* is notable for its richness in seven essential amino acids, with the exception of tryptophan. The total content of essential amino acids in *Anser cygnoides* meat constitutes 45.38% of the overall amino acids present in goose meat, and the lysine content, which is closely associated with food flavor, is as high as 16.34%. This meat is beneficial for preventing cardiovascular and cerebrovascular diseases and is often referred to as the king of green food in the world. This characteristic has become a unique advantage of *Anser cygnoides* compared to other poultry meats.

Recombination technology is a widely utilized method in meat processing (Li & Han, 2014). According to the adhesion mechanism of recombinant meat, recombinant technologies can be classified into enzymatic, chemical and physical processing technologies. These technologies have been widely used in the processing of livestock, poultry and fish meat products. The Food and Agriculture Organization of the United Nations (Food and Agriculture Organization [FAO], 2014) has projected that global meat consumption will double by 2050 due to the increasing population. Over the next decade, a growth rate of 14 % in meat consumption is anticipated (Organisation for Economic Co-Operation and Development [OECD], 2021). Rising income levels and population growth are critical factors driving the demand for meat (Alam et al., 2024). To address the escalating demand for meat in the coming years, enhanced resources for meat processing technology are essential. Consequently, various alternatives have been developed to meet this demand, including cultured meat (Post et al., 2020), hybrid cultured meat (Alam et al., 2024), hybrid meat (Baune et al., 2023), and plant-based meat (Kumari et al., 2023). The process of combining different ingredients to create a new product with high nutritional value is referred to as restructured meat (Polášek et al., 2021). Recombinant meat involves the use of meat scraps generated during raw meat production, which are re-bonded using appropriate food additives and processing technologies to create meat products that resemble the quality and properties of whole meat (Chi, 2008). The fundamental principle of restructuring meat (RM) is to create a mixture of various types of meat, potentially supplemented with plant-based proteins and fiber sources. This combination serves as the foundation for achieving the desired taste, consistency, and nutritional profile of the final product (Patel & Nayak, 2023). TG exhibits strong adhesion properties and can be applied to recombine pork to produce new pork sausage products (Ren et al., 2024). Currently, the synergistic effect of bonding technology and additive processing in recombinant meat production is crucial for enhancing product quality. Studies have evaluated the roles of transglutaminase (TG), gluconolactone (GDL), fibronectin (FS), gelatin, hydrocolloids, phosphates, starch, and cellulose in meat reconstitution. Research on recombinant meat products encompasses the optimization of processing conditions and the incorporation of salt, phosphate, and nitrite to improve quality and consumer acceptability. Additionally, this research includes recombinant protein expression in meat products, the development of low-sodium, high-fiber, and antioxidant-rich options, as well as the application of extrusion-based 3D food printing technology (Samad et al., 2024a, Samad et al., 2024b). In particular, 3D-printed meat analogs using alternative proteins, including plant-based proteins or insects, have been developed in recent years (Chao et al., 2022).

Although research on restructured meat began relatively late in China, the findings from related studies have been successfully implemented in industrial production. Despite the limited research on reassembling goose steak, this study utilizes *Anser cygnoides*, post-liver extraction, as a raw material to maximize its economic value. This paper presents the development of recombination technology for goose steak, along with the optimization of production conditions through single-factor and response surface tests. Ultimately, a goose steak with a compact structure and favorable taste was achieved, aiming to serve as a reference for enhancing the comprehensive utilization value of *Anser cygnoides* meat.

## 2 Experiments and methods

### 2.1 Experimental materials and equipment

*Anser cygnoides* leg meat, *Anser cygnoides* breast meat (Zunrun Sheng Luojie Food Co., Ltd.,Shandong,China) (The best age to take foie gras for *Anser cygnoides* are 12 weeks of age or older, and they start to fill out when they weigh 4-5 kg, and they can weigh up to 8.1–8.5 kg at the time of slaughter); Compound enzyme (including TG enzyme, sodium caseinate, salt,maltodextrin) (Transglutaminase, TG) (Loncote Enzyme Preparation Co., Ltd.,Shandong,China); Haier freezer BD-330WEPTU1 (Haier Special freezer Co., Ltd.,Shandong,China); Quick freezing Test equipment D/DW-100E (Yiyan Experimental equipment Co., Ltd.,Shanghai,China); Electronic balance FA1204N (Jinghai instrument Co., Ltd.,Shanghai,China); Midea Induction cooker C21-SK2103 (Midea Life Appliance Manufacturing Co., Ltd.,Guangdong,C); Texture instrument TMS-PRO*(FTC,America); Color difference instrument SC-10 (Sanen Technology Co., Ltd.,Shenzhen,China); Roll kneading machine 5 L self-made.

### 2.2 Single factor condition optimization

The frozen goose leg and breast meat are naturally thawed at 4°C. Once thawed, the goose meat is separated from fat and fascia, then washed in ice water. After cleaning the surface of the goose meat, the water is drained, and the meat is set aside. The next step involves cutting the goose meat into small pieces, approximately 1 cm^3^. Subsequently, compound enzymes are added at varying percentages, ranging from 0.5% to 4.5% while maintaining a temperature between 0 °C and 4 °C. The mixture is then rolled and kneaded for different durations: 0, 1, 2, 3, and 4h. Following this, the goose meat is wrapped into a cylinder using plastic film and refrigerated at 4 °C for 2, 6, 8, and 10 h before being transferred to frozen storage at −18°C. The cylinder measures 30 cm in length and 10 cm in diameter. For the performance test, the frozen goose steak is removed from the freezer and slowly thawed in the refrigerator at 4 °C prior to testing.

### 2.3 Determination of bond strength

Texture plays a crucial role in determining the mouthfeel and overall sensory experience of meat products (Samad et al., 2024a, Samad et al., 2024b). To replicate the fibrous composition found in conventional cuts, restructured meat (RM) undergoes a rigorous processing method. Following the research methodology outlined by Pietrasik and Gaudette (2014), a sample was extracted and cut into dimensions of 5 cm × 2 cm × 0.4 cm. A tensile test was subsequently conducted using a clip probe. The force arm of the texture analyzer was set to 1000 N, with a test speed of 30 mm/min and pre-test and post-test speeds of 35 mm/min. The test was performed in tension mode, with an inductance of 0.1 N and a fracture distance of 40 mm. The probe ascended to determine the maximum force required to rupture the meat strips, and bond strength was then calculated using a specific formula.

(1) $$\text{Bond strength}/\left(g/{\mathrm{cm}}^{2}\right)=F/S$$

In the formula, F represents the maximum force required to break the meat, while S denotes the cross-sectional area in square centimeters (cm^2^) of the meat.

### 2.4 Determination of full texture

The goose meat steak was swiftly cut into 2.5 cm^3^ cubes, following the Texture Profile Analysis (TPA) methods outlined by Singh et al. (2013). Three parallel experiments were conducted for each sample, and the results were subsequently analyzed. The test parameters included a pretest speed of 50 mm/min, a test speed of 50 mm/min, a post-test speed of 50 mm/min, a target mode of 100 % strain, and a duration of 5s. The analysis focused on hardness, elasticity, cohesion, chewiness, stickiness, and adhesiveness.

### 2.5 Determination of color

The method employed by Kayaardı and Gök (2004) was utilized to measure the luminance (L*), redness (a*), and yellowness (b*) values of the goose steak samples. To operate the CR-400 colorimeter, first ensure that the instrument is calibrated, typically using a standard white reference board. Aim the measuring head of the colorimeter at the surface of the goose steak, maintaining a specific distance and angle to minimize the influence of shadows or direct light. Press the measurement button and record the L*, a*, and b* values. The L* value indicates brightness, the a* value indicates red-greenness, and the b* value indicates yellow-blueness. Three random positions were selected for each sample, and each position was measured three times. The average result was then recorded.

### 2.6 Determination of cooking loss rate

Following the method described by Tian et al. (2016), the sample was removed from the refrigerator and weighed, denoted as M. It was then placed in a cooking bag and heated for 20min in a water bath at 80 °C. After heating, the meat was removed, allowed to cool to room temperature to absorb any surface moisture, and subsequently weighed again, denoted as m.

(2) $$\text{cooking loss rate}\;\left(\%\right)=M-m/M\times 100\%$$

### 2.7 Sensory evaluation

The flavor of restructured meat (RM) plays a crucial role in the culinary experience, as it aims to accurately replicate the taste of traditional meat while potentially introducing additional functional qualities. The interplay of various components, the use of flavor enhancers, and the culinary methods employed collectively shape the final flavor profile (Chen & Cai, 2021). This study investigates techniques to enhance umami, the savory flavor typically associated with meat, in restructured food products to foster positive consumer perceptions. The method outlined by Cofrades et al. (2011) involved cooking the goose in a frying pan with a small amount of butter for approximately 2 min, with 1min allocated to each side. A sensory evaluation was conducted by a panel of 10 graduate students from the School of Food at Jiangsu Ocean University, who assessed various indicators, including color, aroma, taste, texture, and overall acceptability of the products. Each evaluator independently rated the fried samples based on the specified criteria detailed in Table 1.

**Table 1:** Sensory scoring criteria for goose steak.

Evaluation index	Scoring standard		
	13–20	8–13	1–7
Color and luster(20point)	Good color, appetite and luster	Average color, poor appetite, average luster	Poor color, loss of appetite, poor luster
smell(20point)	The meat has a rich flavor and no fishy smell	The meat is lack of flavor and smells fishy	The smell of meat is light and fishy
Taste(20point)	The meat is delicate, chewy and has a good aftertaste	Feel a little firewood, dry	The meat is hard and has little aftertaste
Organizational state(20point)	The meat slices are intact, not loose, compact and elastic	The meat slices are relatively complete and compact in structure	The meat slices are not dense, loose and unshaped
Overall acceptability (20point)	High (like)	Moderate	Low (not accepted)

Note: This study was approved by the ethics committee of Jiangsu Ocean University of Technology, China. All procedures were conducted in compliance with relevant laws and institutional guidelines. All sensory raters agreed and signed consent forms.

The study was approved by the Ethics Committee of the Jiangsu Institute of Marine Technology in China. All procedures adhered to relevant laws and institutional guidelines. All sensory raters consented to participate. There were no significant ethical implications.

### 2.8 Response surface optimization and production

According to the results of the single-factor experiment, the appropriate factor levels were selected, the response surface experiment was conducted, and the process formula was optimized.

### 2.9 Statistical analysis of data

In all experiments, each batch of goose steak was measured three times, and the data were analyzed using the General Linear Model program in the statistical software SPSS version 26.0, with results presented as mean ± standard deviation. The significance of the means was evaluated using a one-way analysis of variance (ANOVA) followed by Duncan's multiple range test. Graphs were created using Origin 2018 software.

## 3 Result and discussion

### 3.1 Effect of adding amount of compound enzyme on the quality of goose steak

**Fig. 1:**
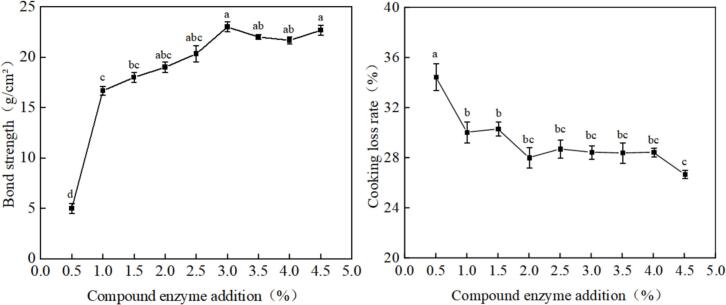
A:effect of complex enzyme addition on enzyme addition on cooking loss rate B:effect of complex enzyme addition on enzyme addition on bond strength.

As illustrated in Fig. 1(A), there is a significant increase in the bond strength of goose steak with rising quantities of the compound enzyme, gradually increasing from 5.0 g/cm^2^ to 23.1 g/cm^2^. The bond strength peaked at a 3 % concentration of the compound enzyme, reaching 23.1 g/cm^2^, which is comparable to that of fresh goose meat at 25.5 g/cm^2^. This enhancement can be attributed to the catalytic action of the transglutaminase (TG) enzyme on acyl groups within goose proteins, facilitating group transfer reactions that result in covalent cross-linking between proteins. This cross-linking and aggregation of goose proteins strengthen the overall structure, thereby enhancing adhesion among minced meat. The role of sodium caseinate in complex enzymes is primarily reflected in the improvement of emulsification properties through the Maillard reaction, the enhancement of solubility and functionality of proteins, and its effectiveness as a substrate for enzyme modification. These properties provide sodium caseinate with a wide range of potential applications in the food industry (Grigorovich et al., 2012).

As illustrated in Fig. 1(B), the cooking loss rate of goose steak exhibited significant variation in response to different amounts of compound enzyme (*P* < 0.05). Specifically, it decreased from 34.45 % to 26.69 %, indicating a pronounced reduction in the cooking loss rate (P < 0.05). The lowest cooking loss rate of 26.69 % was recorded when the amount of compound enzyme was 4.5 %. This reduction in cooking loss rate may be attributed to the cross-linking of the transglutaminase (TG) enzyme and sodium caseinate with goose protein. This cross-linking process forms a protective film around the minced goose meat, effectively trapping water molecules and preventing their escape during cooking, thereby ultimately reducing the cooking loss rate of the product (Sakai et al., 2021).

Table 2 illustrates that the addition of a compound enzyme significantly affects the textural properties of reconstituted goose steak. The hardness of the goose steak notably increased in the treatment group. Previous research by Cao and Zhang (2016) supports this finding, indicating that the inclusion of a compound enzyme enhances the textural properties of reconstituted goose steak. The introduction of the compound enzyme promotes the cross-linking and bonding of minced goose meat, resulting in a denser and firmer tissue structure in goose chops. This improved structure enhances strength during chewing, leading to increases in hardness, elasticity, and chewiness.

**Table 2:** Effect of the addition of complex enzymes on the textural properties.

Adding amount of compound enzyme /%	Bond strength/g/cm^2^	Hardness/N	Elasticity/mm	Cohesion	Chewiness/mJ	Gluing property/N
0.5	5.0 ± 0.8d	235.2 ± 8.89c	3.21 ± 0.26c	0.22 ± 0.051c	183.79 ± 8.26a	42.42 ± 7.50a
1	16.7 ± 1.2c	241.43 ± 0.88c	3.68 ± 0.31c	0.25 ± 0.016bc	214.82 ± 33.62b	43.63 ± 7.27a
1.5	18.1 ± 0.8bc	258.7 ± 12.41bc	3.72 ± 0.33c	0.24 ± 0.016bc	217.19 ± 33.77 cd	49.17 ± 10.25a
2	19.0 ± 0.8abc	268.5 ± 11.68ab	3.87 ± 0.11c	0.25 ± 0.014bc	251.23 ± 18.38bc	53.41 ± 6.96a
2.5	20.3 ± 3.2abc	281.7 ± 13.80ab	4.76 ± 0.44b	0.26 ± 0.021bc	276.99 ± 7.73bcd	57.13 ± 3.21a
3	23.1 ± 1.6a	285.2 ± 7.78a	5.28 ± 0.12b	0.25 ± 0.012bc	301.19 ± 29.41de	58.54 ± 0.80a
3.5	22. ± 0.81ab	269.93 ± 11.70ab	4.96 ± 0.34b	0.29 ± 0.031a	265.62 ± 8.05ef	56.7 ± 2.0a
4	21.7 ± 2.5ab	276.3 ± 13.79ab	5.16 ± 0.08b	0.3 ± 0.016a	320 ± 10.61ef	59.6 ± 4.83a
4.5	22.7 ± 3.3a	277.3 ± 8.97ab	6.30 ± 0.96a	0.29 ± 0.017b	389.95 ± 23.55f	63.1 ± 9.45a

Note: a-f indicates that there is a significant difference between the average values of different data in the same column (*P* < 0.05).

The effect of the amount of compound enzyme on the color of goose steak is illustrated in Table 3. The L* value in the table represents the luminance of the steak, indicating its whiteness, while the a* value denotes redness, and the b* value reflects yellowness. A higher a* value corresponds to a more pronounced reddish color, whereas a higher b* value signifies increased yellowness. The addition of the compound enzyme significantly influenced the color of the product (*P* < 0.05). Notably, the L* value, a* value, and b* value of goose steak all exhibited significant decreases. Specifically, the L* value declined from 51.47 to 42.79, the a* value decreased from 13.73 to 7.78, and the b* value fell from 12.66 to 7.87.

**Table 3:** Effect of complex enzyme addition on color.

Adding amount of compound enzyme/%	L*	a*	b*
0.5	51.47 ± 0.59a	13.73 ± 0.44a	12.66 ± 1.04a
1	48.75 ± 0.87ab	13.28 ± 0.97a	12.04 ± 1.11a
1.5	48.91 ± 3.42ab	11.47 ± 1.59abc	11.56 ± 0.43ab
2	46.07 ± 0.17bcd	10.2 ± 1.39bcd	9.96 ± 1.32bc
2.5	44.31 ± 1.79cde	11.85 ± 0.64ab	11.27 ± 0.94ab
3	47.32 ± 2.53bc	11.33 ± 2.36abc	12.22 ± 0.48a
3.5	41.24 ± 1.17e	8.68 ± 1.22 cd	8.45 ± 0.89 cd
4	42.22 ± 1.58de	9.78 ± 0.45bcd	9.78 ± 0.45bc
4.5	42.79 ± 0.52de	7.87 ± 0.46d	7.87 ± 0.46d

Note: a-f indicates that there is a significant difference between the average values of different data in the same column (P < 0.05).

**Fig. 2:**
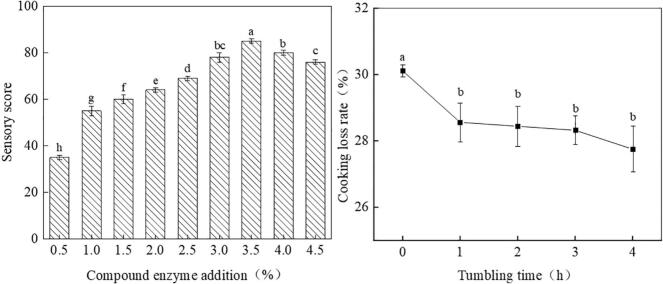
A:effect of complex enzyme addition on sensory score B:effect of tumbling time on cooking loss.

As illustrated in Fig. 2(A), the sensory score of goose steak initially increased with the addition of the compound enzyme, before subsequently declining. Notably, the sensory score reached its lowest point with an addition of 0.5 %. This minimal addition was insufficient to adequately cross-link the minced goose meat, resulting in weak bonding within the steak, a subpar taste experience, and a reduced sensory score. Conversely, at a 3.5 % addition of the compound enzyme, the sensory score peaked, resulting in a goose steak with firm adhesion and a pleasing, chewable texture. However, exceeding the 3.5 % threshold led to an overly dense steak, compromising both meat quality and taste, which ultimately resulted in a decline in the sensory score.

### 3.2 Effect of tumbling time on the quality of goose steak

Fig. 2(B) illustrates the impact of tumbling time on the cooking loss rate of goose steak. The cooking loss rate for the rolling group was significantly lower than that of the non-rolling group (*p* < 0.05). The rolling treatment improved the water retention capacity of the meat.

**Fig. 3:**
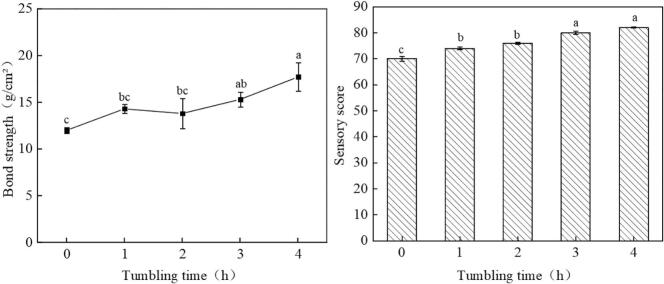
A:effect of tumbling time on bond strength B:effect of tumbling time on sensory score.

The effect of tumbling time on the bond strength of goose steak is illustrated in Fig. 3(A). The study demonstrated that the bond strength of the rolling group was significantly greater than that of the non-rolling group (p < 0.05). Furthermore, tumbling time was shown to exert a significant influence on the bond strength of goose steak (p < 0.05). Notably, the highest bond strength of 17.7 ± 3.1 g/cm^2^ was recorded at a tumbling time of 4 h. This observation may be attributed to the gradual dissolution of proteins in the goose meat during the rolling process, which facilitates interactions with the transglutaminase (TG) enzyme, sodium caseinate, and other adhesives. These interactions promote cross-linking, thereby enhancing the bonding strength of the goose steak.

The effect of tumbling time on the color of goose steak is presented in Table S1, where L*, a*, and b* represent the luminance value, red value, and yellow value of the meat sample, respectively. The results indicate that tumbling time did not significantly affect the color of the product (*P* > 0.05), with only minimal changes observed in the L*, a*, and b* values of the goose steak. Previous research suggests that tumbling may enhance the formation rate of high‑iron myoglobin, which can lead to yellowing of the meat (Yuan, 2011), resulting in a decrease in the b* value.

Table S2 illustrates that the texture properties of recombinant goose steak are influenced by tumbling time. While the hardness of the goose steak in the treatment group did not exhibit significant changes, a discernible trend toward increased hardness was observed. Additionally, the elasticity, chewiness, and gluability of the goose steak improved with longer tumbling times. This enhancement can be attributed to the repeated beating during rolling and kneading, which softens the goose meat tissue and consequently increases the contact area between the meat and adhesive. This improved contact promotes better adhesion and enzymatic reactions. A study by Bombrun and Gatellier (2015) noted that rolling can disrupt muscle fibers and soften the tissue structure of raw meat, which aligns with the findings of this experiment.

The effect of tumbling time on sensory scores is illustrated in Fig. 3(B). This figure demonstrates that as tumbling time increases, the sensory score of goose steak exhibits a positive trend, peaking at a tumbling duration of 4 h. The tumbling process contributes to the loosening of goose tissue, facilitating enzyme reactions that promote protein cross-linking. Furthermore, it enhances the penetration of spices and pickling solutions, reduces curing time, and improves the overall flavor of goose steak (Yu & Zhao, 2022).

### 3.3 Effect of molding time on the quality of goose steak

**Fig. 4:**
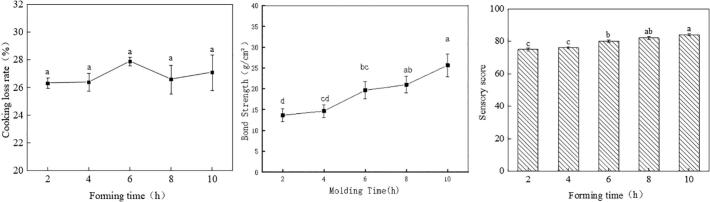
A:effect of forming time on cooking loss rate B:effect of the forming time on the bond strength C:effect of reaction time on sensory score.

**Fig. 5:**
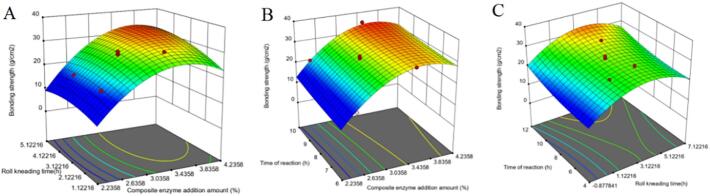
A:effect of enzyme addition and tumbling time on bonding strength B:effect of enzyme addition and setting time on bond strength C:effect of enzyme addition and setting time on bond strength.

The effect of molding time on the cooking loss rate of goose steak is illustrated in Fig. 4(A). The experimental findings indicate that molding duration does not significantly influence the cooking loss rate of goose steak (*p* > 0.05), suggesting that molding time does not affect the water retention of the meat. The impact of molding time on the bond strength of goose steak is presented in Fig. 4(B). The results reveal a significant correlation between molding time and bond strength. Specifically, at a molding time of 2 h, the bond strength is at its lowest, measuring 13.7 ± 1.2 g/cm^2^. Nevertheless, the goose steak remains tightly bound, with the slices retaining their structural integrity. In contrast, a molding time of 10 h results in the highest bond strength, recorded at 25.6 ± 3.8 g/cm^2^. This increase can be attributed to the time required for the cross-linking of the transglutaminase (TG) enzyme reaction, commonly referred to as enzyme reaction time. In a controlled environment, a longer reaction time facilitates a more thorough interaction, leading to a more favorable outcome. (See Fig. 5.)

Table S3 illustrates the effect of molding time on the color of goose steak, with L*, a*, and b* representing the luminance value, red value, and yellow value of the meat sample, respectively. The results indicate that molding time did not significantly affect the color of the product (*P* > 0.05). Specifically, the L* value of goose steak remained relatively constant, while both the a* and b* values exhibited a slight decrease.

Table S4 illustrates that the texture properties of recombinant goose steak are influenced by molding time. Notably, the hardness of goose steak shaped for 10 h was significantly greater than that of goose steak shaped for 2 h (*P* < 0.05). This observation can be attributed to the necessity of a specific duration for enzyme reactions to occur. In a controlled environment, a longer reaction time promotes a more complete reaction, resulting in enhanced bonding and increased hardness. Conversely, the elasticity, cohesion, chewiness, and gluability of goose steak did not show significant changes with an increase in setting time. The effect of molding time on the sensory score is illustrated in Fig. 4(C).

The figure clearly indicates that the sensory score of goose steak gradually increases with longer setting times. The highest sensory score was recorded at a setting time of 10 h. Molding time significantly influences the bonding of goose steak; the tighter bonding achieved with extended setting times contributes to a subtle enhancement in flavor.

### 3.4 Response surface analysis

Based on the results of the single-factor test, the amounts of compound enzyme added, tumbling time, and molding time were treated as response variables, while bond strength was considered the response value. A response surface test was conducted utilizing the principles of the Box-Behnken design, implemented through Design Expert 8.0 software. Three distinct levels were selected for each variable. The experimental scheme and results are presented in Table S5.

The quadratic multinomial regression model equation for goose steak bond strength, derived from the analysis of the response surface, incorporates the effects of compound enzyme, tumbling time, and molding time. The equation is expressed as y = 27.60 + 7.00 A + 1.00B + 1.50C + 0.50AB - 2.55A^2^–0.50B^2^ + 0.45C^2^.

According to the variance analysis table presented in Table S6, the *p*-value of the model is 0.0009, indicating a good fit. The p-value of the misfit term is 0.7222, which is greater than 0.05, suggesting that it is not statistically significant. This further confirms that the model is not misfitting and that the selection of the model is appropriate. Based on the literature, an R^2^ value greater than 0.75 is considered acceptable for the model (Chauhan & Gupta, 2004). The correlation coefficient for this model is 0.9508, indicating a relatively reliable model that can be used to predict test results. In the first term, the order of the effects of compound enzyme addition (A), tumbling time (B), and molding time (C) on bond strength was found to be A > C > B. The addition of compound enzyme (A) had a significant effect on bond strength (*p* < 0.05).

Fig. 4(A) demonstrates that bond strength increases with both the addition of the compound enzyme and the duration of tumbling time. Furthermore, bond strength also rises with greater compound enzyme addition and extended setting time; however, the interaction observed is less pronounced than that depicted in Fig. 4(B). Fig. 4(C) indicates that as tumbling time and forming time increase, the surface transitions from smooth to steep, suggesting that the interaction between tumbling time and forming time significantly influences bond strength. Notably, the amount of compound enzyme exhibits a strong interaction with other factors, which is consistent with the results of the analysis of variance.

The optimization of goose steak production was achieved using Design-Expert 11 to solve the regression equation. The optimal technological conditions identified for goose steak production were as follows: the amount of compound enzyme was set at 3.5 %, the tumbling time was 4 h, the setting time was 10 h, and the bonding strength measured 34.950 g/cm^2^. The verification of the optimization process involved applying these refined conditions for goose steak production, which are as follows: the compound enzyme amount remained at 3.5 %, the tumbling time was maintained at 4 h, and the setting time was 10 h. Under these conditions, the bond strength of the goose steak reached 35.2 g/cm^2^, with the deviation between the target parameter test value and the model's predicted value being less than 1 %. This indicates that the model exhibits a high degree of accuracy and can effectively simulate the goose steak production process.

## 4 Conclusion

The study utilized ligated *Anser cygnoides* as a primary ingredient for the production of recombinant goose steak, aiming to maximize the economic value of *Anser cygnoides*. The effects of compound enzyme addition, tumbling time, and molding time on the recombinant goose steak were evaluated based on various criteria, including bond strength, cooking loss rate, color, hardness, elasticity, cohesion, chewiness, stickiness, adhesiveness, and sensory score, and a new process of reconstituted Landess goose steak was established. The proposed method not only improves the product quality and nutritional value of reconstituted goose steak, but also promotes the technological progress and innovation in the field of meat simulation products. By applying these research findings, consumer demand for healthy, tasty, and sustainable food can be better met. Post-frying, the shape of the recombinant goose steak remained intact, earning an excellent sensory score.

## Author contribution

Conceptualization, Shengfu Li and Junjie Zhang; methodology, Saisai Zhang and Shengfu Li; software, Saisai Zhang; validation, Saisai Zhang and Linwu Zhuang; formal analysis, Saisai Zhang; investigation, Saisai Zhang; resources, Shengfu Li and Junjie Zhang; data curation, Saisai Zhang and Hanrui Wang; writing—original draft preparation, Saisai Zhang and SiYuan Li; writing—review and editing, Saisai Zhang and Hanrui Wang; visualization, Saisai Zhang; supervision, Junjie Zhang; project administration, Shengfu Li; funding acquisition, Junjie Zhang. All authors have read and agreed to the published version of the manuscript.” Please turn to the CRediT taxonomy for the term explanation. Authorship must be limited to those who have contributed substantially to the work reported.

## Funding

This research was supported by A Project Funded by the 10.13039/501100012246Priority Academic Program Development of Jiangsu Higher Education Institutions (PAPD), (“ginger urge to drink”, −research of ginger wine with sea features, Fund Number: SY202411641638006; Preparation and study of decellularized matrix of fish skin, Fund Number: SZ202211641638001).

## CRediT authorship contribution statement

**Saisai Zhang:** Writing – review & editing, Writing – original draft, Validation, Software, Methodology, Investigation, Formal analysis, Data curation, Conceptualization. **Hanrui Wang:** Data curation. **SiYuan Li:** Writing – original draft. **Junjie Zhang:** Supervision, Resources, Funding acquisition, Conceptualization. **Linwu Zhuang:** Validation. **Shengfu Li:** Resources, Project administration, Methodology, Conceptualization.

## Declaration of competing interest

The authors declare that they have no known competing financial interests or personal relationships that could have appeared to influence the work reported in this paper.

## Data Availability

The authors do not have permission to share data.

## Appendix A Supplementary data

Supplementary material 1 Image 1

Supplementary material 2 Image 2
