# Supplementary material for: Research on a new process of reconstituted landess goose steak
Source: Food Chem X. 2024 Dec 30;25:102118. doi: 10.1016/j.fochx.2024.102118 (PMC11758398; doi:10.1016/j.fochx.2024.102118)
Supplement: Supplementary file 1 — Supplementary material 1 [file mmc1.docx]

**Table S1.** Effect of tumbling time on the colour

| Rolling and kneading time/h | L* | a* | b* |
| --- | --- | --- | --- |
| 0 | 47.14±2.78a | 13.4±1.04a | 12.12±1.21a |
| 1 | 42.11±4.49a | 12.65±0.50a | 10.01±1.43ab |
| 2 | 47.32±2.53a | 11.33±2.36a | 12.22±0.48a |
| 3 | 43.73±0.37a | 10.69±0.61a | 9.24±0.53b |
| 4 | 47.55±1.41a | 13.49±0.32a | 11.14±0.72ab |

Note: a -f indicates that there is a significant difference between the average values of different data in the same column (P < 0.05).

**Table S2.** Effect of rolling time on texture characteristics

| **Rolling and kneading time/%** | **Bond strength/g/cm2** | **Hardness/N** | **Elasticity/mm** | **Cohesion** | **Chewiness/mJ** | **Gluing property/N** |
| --- | --- | --- | --- | --- | --- | --- |
| 0 | 12±0.3c | 219.51±4.30ab | 3.13±0.69b | 0.30±0.035a | 204.67±27.69b | 66.65±5.85ab |
| 1 | 14.3±0.5bc | 205.73±11.83b | 4.55±0.06a | 0.30±0.00a | 254.11±44.66ab | 55.73±9.16c |
| 2 | 13.8±1.6bc | 228.50±8.39a | 4.46±0.19a | 0.29±0.012a | 301.19±29.41a | 58.54±0.80bc |
| 3 | 15.3±0.8ab | 231.81±14.63a | 4.29±0.12a | 0.29±0.026a | 299.45±25.22a | 68.57±2.94a |
| 4 | 17.7±3.1a | 226.70±12.69a | 4.39±0.31a | 0.29±0.008a | 290.55±22.56a | 66.11±2.11ab |

Note: a-f indicates that there is a significant difference between the average values of different data in the same column (P < 0.05)

**Table S3.** Effect of reaction time on the colour

| Reaction time/h | L* | a* | b* |
| --- | --- | --- | --- |
| 2 | 43.47±0.34a | 9.32±0.57a | 11.70±0.59a |
| 4 | 42.52±1.50a | 8.93±0.27ab | 11.24±0.35ab |
| 6 | 40.81±1.24a | 6.97±0.18c | 9.36±1.26c |
| 8 | 41.06±1.41a | 8.17±0.22b | 9.87±0.45bc |
| 10 | 40.82±1.36a | 8.44±0.28b | 9.86±0.25bc |

Note: a-f indicates that there is a significant difference between the average values of different data in the same column (P < 0.05).

**Table S4.** Effect of reaction time on the textural characteristics

| **Forming time/%** | **Bond strength/g/cm2** | **Hardness/N** | **Elasticity/mm** | **Cohesion** | **Chewiness/mJ** | **Gluing property/N** |
| --- | --- | --- | --- | --- | --- | --- |
| 2 | 13.7±1.2d | 235.73±39.74b | 5.11±0.57a | 0.3±0.02a | 355.45±51.54a | 69.56±5.77a |
| 4 | 14.7±1.2cd | 228.63±15.81b | 5.3±0.74a | 0.26±0.01a | 324.87±79.79a | 60.53±7.27a |
| 6 | 19.6±3.2bc | 250.93±26.63ab | 5.11±0.26a | 0.28±0.04a | 361.48±49.41a | 70.46±5.76a |
| 8 | 21±1.6ab | 231.83±26.91b | 5.24±0.26a | 0.29±0.01a | 352.17±52.69a | 67.2±9.73a |
| 10 | 25.6±3.8a | 303.13±6.16a | 5.21±0.31a | 0.3±0.01a | 415.88±73.34a | 91.1±4.38b |

Note: a-f indicates that there is a significant difference between the average values of different data in the same column (P < 0.05).

**Table S5.** Response surface scheme design and test results

| **Std** | **run** | **AEnzyme addition%** | **BRolling time/h** | **CForming time/h** | **Bond strength/g/cm^2^** |
| --- | --- | --- | --- | --- | --- |
| 15 | 1 | 3 | 3 | 8 | 28 |
| 4 | 2 | 3.5 | 4 | 8 | 32 |
| 2 | 3 | 3.5 | 2 | 8 | 30 |
| 1 | 4 | 2.5 | 2 | 8 | 18 |
| 8 | 5 | 3.5 | 3 | 10 | 35 |
| 9 | 6 | 3 | 2 | 6 | 25 |
| 5 | 7 | 2.5 | 3 | 6 | 16 |
| 10 | 8 | 3 | 4 | 6 | 28 |
| 17 | 9 | 3 | 3 | 8 | 29 |
| 11 | 10 | 3 | 2 | 10 | 27 |
| 3 | 11 | 2.5 | 4 | 8 | 18 |
| 16 | 12 | 3 | 3 | 8 | 28 |
| 13 | 13 | 3 | 3 | 8 | 24 |
| 7 | 14 | 2.5 | 3 | 10 | 20 |
| 12 | 15 | 3 | 4 | 10 | 30 |
| 14 | 16 | 3 | 3 | 8 | 29 |
| 6 | 17 | 3.5 | 3 | 6 | 31 |

**Table S6.** Analysis of variance for the regression equation parameters

| **Data source** | **Sum of squares** | **Degree of freedom** | **Mean square** | **F** | **p** | **Significance** |
| --- | --- | --- | --- | --- | --- | --- |
| Model | 448.68 | 9 | 49.85 | 15.04 | 0.0009 | significant |
| A-Adding amount of compound enzyme | 392.00 | 1 | 392.00 | 118.28 | < 0.0001 | * * |
| B-Rolling time | 8.00 | 1 | 8.00 | 2.41 | 0.1642 |  |
| C-Forming time | 18.00 | 1 | 18.00 | 5.43 | 0.0526 |  |
| AB | 1.0000 | 1 | 1.0000 | 0.3017 | 0.5999 |  |
| AC | 0.0000 | 1 | 0.0000 | 0.0000 | 1.0000 |  |
| BC | 0.0000 | 1 | 0.0000 | 0.0000 | 1.0000 |  |
| A² | 27.38 | 1 | 27.38 | 8.26 | 0.0238 | * |
| B² | 1.27 | 1 | 1.27 | 0.3843 | 0.5549 |  |
| C² | 0.8526 | 1 | 0.8526 | 0.2573 | 0.6276 |  |
| Residual error | 23.20 | 7 | 3.31 |  |  |  |
| Misfit term | 6.00 | 3 | 2.00 | 0.4651 | 0.7222 | not significant |
| Pure error | 17.20 | 4 | 4.30 |  |  |  |
| Total difference | 471.88 | 16 |  |  |  |  |

Note: * * indicates extremely significant difference (p < 0.01), * indicates significant difference (p < 0.05)

**Table S7.** Effect of reaction time on steaming loss

| **Forming time/h** | **2** | **4** | **6** | **8** | **10** |
| --- | --- | --- | --- | --- | --- |
| Cooking loss rate/% | 26.33±0.30a | 26.39±0.53a | 27.91±0.24a | 26.61±1.66a | 27.11±1.86a |

Note: a-f indicates that there is a significant difference between the average values of different data in the same row (P < 0.05).
