# Supplementary material for: Research on a new process of reconstituted landess goose steak
Source: Food Chem X. 2024 Dec 30;25:102118. doi: 10.1016/j.fochx.2024.102118 (PMC11758398; doi:10.1016/j.fochx.2024.102118)
Supplement: Supplementary file 2 — Supplementary material 2 [file mmc2.pdf]

Ethical statement

NO. Oct20241015

An ethical statement on *Research on the process of reconstituted Landess goose steak*. This study was approved by the ethics committee of Jiangsu Ocean University of Technology, China. All procedures were conducted in compliance with relevant laws and institutional guidelines. All sensory raters agreed and signed consent forms.

Ethics committee of Jiangsu Ocean University of Technology

October 10, 2024
